# Glomeruloid microvascular proliferation is associated with p53 expression, germline *BRCA1* mutations and an adverse outcome following breast cancer

**DOI:** 10.1038/sj.bjc.6601195

**Published:** 2003-09-09

**Authors:** J R Goffin, O Straume, P O Chappuis, J-S Brunet, L R Bégin, N Hamel, N Wong, L A Akslen, W D Foulkes

**Affiliations:** 1 Tufts University Department of Medicine, Division of Hematology/Oncology, Tufts-New England Medical Center, 750 Washington St., Tufts-NEMC #245 Boston, MA 02111; 2 Department of Pathology, The Gade Institute, Haukeland University Hospital, N-5021 Bergen, Norway; 3 Department of Human Genetics, McGill University, Montréal, Québec, Canada; 4 Research Institute of the McGill University Health Centre, McGill University, Montreal, Québec, Canada H2W 1S6; 5 Program in Cancer Genetics, McGill University, Montreal, Quebec, Canada H2W 1S6; 6 Algorithme Pharma, Montreal, Québec, Canada H7V 4B4; 7 Department of Surgery, McGill University, Montréal, Québec, Canada; 8 Department of Pathology, McGill University, Montréal, Québec, Canada; 9 Cancer Prevention Center, Sir MB Davis-Jewish General Hospital, McGill University, Montreal, Québec, Canada H2W 1S6; 10 Department of Medicine, McGill University, Montréal, Québec, Canada

**Keywords:** hereditary breast cancer, angiogenesis, factor VIII, chemotheraphy, survival

## Abstract

Glomeruloid microvascular proliferation (GMP) in breast cancer independently adversely affected survival (relative risk 1.9, 95% CI: 1.2–3.0), particularly among women who received adjuvant chemotherapy (10-year survival 27 *vs* 69%, *P*=0.0003), and was significantly associated with p53 overexpression and *BRCA1* germline mutations. The presence of GMP may influence treatment decisions.

Angiogenesis is under intense study both for its prognostic value and for the potential therapeutic value of interfering with angiogenic pathways. In breast cancer, both higher vascular endothelial growth factor (VEGF) levels (Blackwood and Weber, 1998; Linderholm *et al*, 2000) and increased microvessel density (MVD) (Weidner *et al*, 1991, 1992; de Jong *et al*, 2000) are associated with a poorer prognosis. Recent microarray studies have also identified an association between the expression of genes involved in angiogenesis, such as VEGF, and poor prognosis following breast cancer (van't Veer *et al*, 2002). Furthermore, the proangiogenic genes *COL4A1* and *ECGF1* are overexpressed in breast cancers found in *BRCA1* mutation carriers, although their prognostic value in such women is unknown (van't Veer *et al*, 2002). Here, we describe our results with a morphological marker of prognosis of potential importance in the management of women who carry germline *BRCA1* mutations. Glomeruloid microvascular proliferations (GMPs) (Figure 1

**Figure 1:**
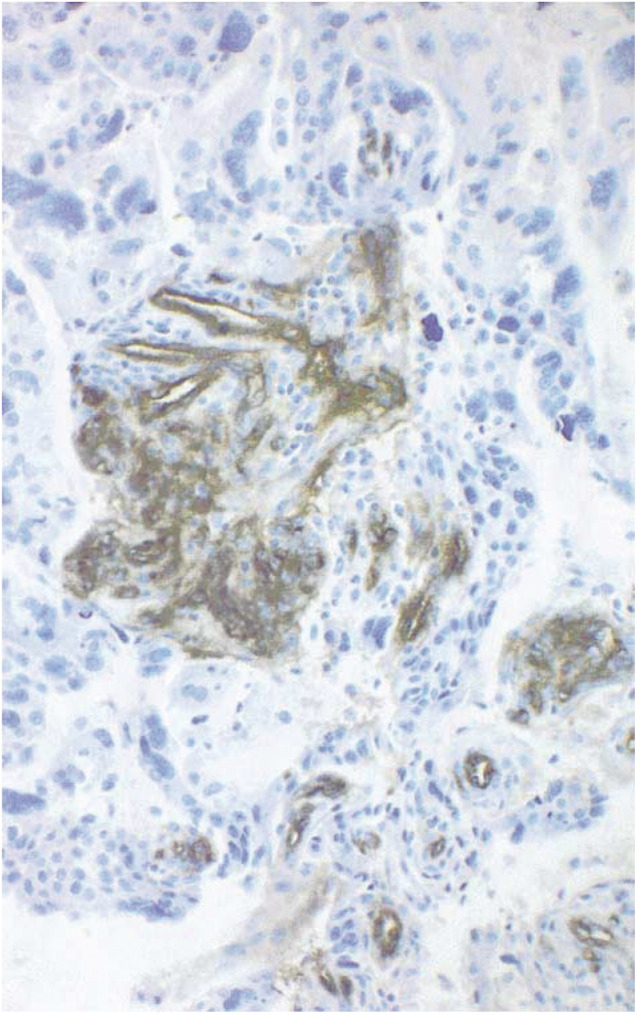
A GMP from a *BRCA1:5382insC*-related breast cancer is shown.

) are focal proliferative buddings of vascular endothelial cells resembling a renal glomerulus. Glomerular microvascular proliferation has been predominantly associated with glioblastoma multiforme (Wesseling *et al*, 1998), the most aggressive form of glioma. They can be produced in athymic mice by overexpressing VEGF using an adenovirus vector (Sundberg *et al*, 2001). Recent evidence suggests that these structures may be present and prognostically useful in other tumour types, including breast cancer (Straume *et al*, 2002). The work presented here is an expansion of the breast cancer data presented by Straume *et al*, and focuses on two new aspects: (1) the inter-relationship between GMP, p53 overexpression and *BRCA1/2* status and (2) the effect of GMP on prognosis in the presence or absence of adjuvant chemotherapy.

## MATERIALS AND METHODS

A total of 292 consecutive Ashkenazi Jewish women aged 65 years or less with primary nonmetastatic breast cancer diagnosed at one Montreal institution between 1980 and 1995 were assessed. Sufficient follow-up and tissue were available for 251 subjects. Following ethics committee approval, specimens were evaluated by one pathologist (LR Bégin) using conventional methods. Accumulation of p53 protein was detected by immunohistochemistry as previously described (Yuan *et al*, 1999). Pathology blocks from all women were tested for founder *BRCA1* mutations (185delAG, *n*=18; 5382insC, *n*=10) and *BRCA2* mutation (6174delT, *n*=8) that are common in this population, using established techniques (Foulkes *et al*, 1997).

Staining of endothelial cells by Factor-VIII (A-0082, Dako, Copenhagen) was performed on formalin-fixed and paraffin-embedded archival material as previously published (Straume and Akslen, 2001). The presence of GMP was recorded by the finding of focal glomerulus-like aggregates of closely associated and multilayered factor-VIII positive endothelial cells. Glomerular microvascular proliferations consisted of 15–100 cells. Lumen formation was not necessary for the aggregates to be counted as GMPs. Tangentially sectioned normal vessels, or nonspecific Factor-VIII positivity in stromal components, were excluded. Glomerular microvascular proliferations were categorised as being absent (group 0), rare (not more than one per high-power field (HPF), group 1), or greater than 1 per HPF (group 2). Microvascular density was calculated as the mean number of stained vessels in 10 high-power fields (× 400).

Molecular, pathological and clinical assessments were collected in a mutually blinded manner in a retrospective cohort approach. Subject characteristics were compared using Wilcoxon, *t*-test, and Fisher's exact testing, with trends in increasing odds ratios being assessed by Cochran–Armitage's test. Differences in breast cancer-specific survival were calculated using the method of Kaplan and Meier. The Cox proportional hazards model was used to assess prognostic factors.

## RESULTS

In all, 43 breast cancers (17%) had one or more GMP, with 36 tumours in group 1 and seven in group 2. Their presence was associated with higher nuclear grade (*P* for trend <0.0001), oestrogen receptor (ER) negativity (OR 4.7, 95% CI: 2.3–9.6), p53 immunohistochemical positivity (OR 4.1, 95% CI: 2.0–8.2), and germline *BRCA1* mutations (odds ratio (OR) 2.6, 95% CI: 1.1–6.3), but not tumour size, axillary nodal status, microvascular density (MVD) or germline *BRCA2* mutations (Table 1

**Table 1:** Patient characteristics

**Characteristic^a^**	**Subjects with GMP (percent) *n*=43**	**Subjects without GMP (percent) *n*=208**	***P*-value**
Age, median (range)	50.1 (31.6–65.9)	53.2 (26.5–65.3)	0.57
Tumour size, median (cm) (238)	2.0	1.8	0.15
*Nuclear grade (249)*			
1	2 (4)	61 (30)	
2	15 (35)	84 (41)	<0.0001^b^
3	26 (60)	61 (30)	
*Oestrogen receptor (247)*			
Positive	14 (33)	144 (70)	
Negative	28 (67)	61 (30)	<0.0001
*Lymph node status (228)*			
Positive	17 (44)	90 (48)	0.73
Negative	22 (56)	99 (52)	
*Microvascular density (251)*			
Median (range)	112.5 (43.8-306.3)	115.6 (37.5-393.8)	0.27
*BRCA-carrier status*			
*BRCA1* carrier	9 (21)	19 (9)	0.04
*BRCA2* carrier	1 (2)	7 (3)	0.99
Non-carrier	33 (77)	182 (88)	
*p53 IHC (245)*			
Positive	21 (50)	40 (20)	
Negative	21 (50)	163 (80)	0.0001

GMP=glomeruloid vascular proliferation; IHC=immunohistochemistry.

a Number in parenthesis indicates cases with available data.

b For trend.

). There was no relationship between higher GMP grouping and higher MVD or *BRCA1* mutation type.

There were 65 breast cancer deaths in this series of women at 10 years follow-up. Kaplan–Meier survival analysis showed that 50.3% of women with GMP died of breast cancer over this period, whereas the mortality was 25.7% for those with no identified GMP (*P*=0.0003). Microvascular density was not significantly associated with a worse prognosis (*P*=0.47). In a Cox proportional hazards model, the presence of GMP (defined continuously) was associated with a poor prognosis (relative risk (RR) 1.9, 95% CI: 1.2–3.0) as was positive lymph node status (RR 2.3). Nuclear grade (RR 1.6) and negative ER status (RR 1.7) were of borderline significance, while tumour size, p53 positivity and carrier status did not achieve significance (Table 2

**Table 2:** Cox proportional hazards model for breast cancer specific mortality

	**Univariate analysis**		**Multivariate analysis (*n*=247)**	
**Variable**	**RR (95% CI)**	***P*-value**	**RR (95% CI)**	***P*-value**
*Tumour size* (cm)				
<2	1.0		1.0	
⩾2	2.8 (1.6-4.9)	0.0002	1.6 (0.9-3.0)	0.1
Nuclear grade^a^	2.5 (1.7-3.5)	0.0001	1.6 (1.03-2.4)	0.04

*ER status*				
Positive	1.0		1.0	
Negative	3.0 (1.8-4.8)	0.0001	1.7 (0.95-3.0)	0.07

Lymph nodes				
Negative	1.0		1.0	
Positive	2.5 (1.5-4.4)	0.0007	2.3 (1.3-3.4)	0.004

*Mutation carrier status*				
Non-carriers	1.0		1.0	
*BRCA1* carriers	1.7 (0.9-3.4)	0.1		
*BRCA2* carriers	1.9 (0.6-6.2)	0.3		
*BRCA1/BRCA2* carriers	1.8 (0.96-3.2)	0.07^b^	1.1 (0.6-2.0)	0.8^b^

*p53 IHC*				
Negative	1.0		1.0	
Positive	2.5 (1.5-4.1)	0.0003	1.3 (0.7-2.2)	0.4

GMP^a^	2.4 (1.6-3.5)	0.0001	1.9 (1.2-3.0)	0.006

ER=oestrogen receptor; GMP=glomeruloid microvascular proliferation; IHC=immunohistochemistry; MVD=microvascular density. The model was adjusted for cases with missing tumour size (*n*=13) and missing lymph node status (*n*=23).

a As a continuous variable.

b *BRCA1/BRCA2* carriers combined compared with non-carriers.

). Among women treated with adjuvant chemotherapy, the presence of GMP was an indicator of poor prognosis (10-year survival 27 *vs* 69%, *P*=0.0003), while among women not treated with chemotherapy, no statistically significant difference in survival was seen on the basis of GMP status (10-year survival 75 *vs* 79%, *P*=0.4).

## DISCUSSION

This study is the first to demonstrate that GMP is associated with p53 expression and the presence of germline *BRCA1* mutations and it suggests that the presence of GMP is an independent risk factor for death from breast cancer comparable in magnitude to conventional prognostic factors (RR 1.9).

Notably, GMP was not associated with a higher MVD, and the latter was not prognostic for poor survival in our cohort of patients. Vascular endothelial growth factor is implicated in the genesis of both GMP (Sundberg *et al*, 2001) and increased MVD (De Paola *et al*, 2002), but the lack of association between GMP and MVD suggests that their developmental pathways may differ. In our cohort, p53 expression was associated with the presence of GMP, but not with increased MVD (*P*=0.8), the latter being consistent with the literature (Tas *et al*, 2000). Functional p53 impedes angiogenesis through the regulation of VEGF transcriptional factors Sp1 (Mandlekar and Kong, 2001) and the HIF-1*α* subunit (Ravi *et al*, 2000), as well as by upregulating thrombospondin-1 expression (Dameron *et al*, 1994). Mutated p53 may be one pathway by which a neovascular phenotype associated with GMP (but not MVD) formation is promoted.

Our data suggest that GMP is associated with p53 expression and *BRCA1* germline mutations and that all three of these factors may be associated with a worse survival (for details of the p53-BRCA1 relationship, see Goffin *et al*, 2003). Interestingly, patients who were treated with adjuvant chemotherapy had a poorer outcome if their tumours demonstrated GMP. Glomerular microvascular proliferation was highly significantly associated with p53 expression (*P*=0.0001), a protein partly responsible for inducing apoptosis in chemotherapy-treated cells and thus potentially responsible for diminished responsiveness to chemotherapy (Fisher, 2001). Opposed to this is the association of GMP with *BRCA1* mutations and higher nuclear grade, both of which appear to increase tumour responsiveness to chemotherapy (Chappuis *et al*, 2002;Wang *et al*, 2002). In the present study, higher nuclear grade (RR 1.9, *P*=0.02) and age < 50 years (RR 5.6, *P*=0.0001) were associated with an increased likelihood of a woman receiving adjuvant chemotherapy, while other factors were not significant on multivariate analysis. The apparent contradiction in associations and chemoresponsiveness is likely a product of the interplay of several response mitigating pathways and the incomplete association between measured factors.

There is evidence that *BRCA1*-related breast cancers have a distinct profile on microarray analysis (van't Veer *et al*, 2002) and that these cancers have a distinctive spectrum of *TP53* mutations (Greenblatt *et al*, 2001). Along with evidence that *BRCA1* is important in global nucleotide excision repair (Hartman and Ford, 2002), these data hint that *BRCA1* mutations induce a genetic profile of which p53 expression and GMP are but two manifestations, with several factors influencing both prognosis and response to treatment. However, the role of *BRCA1* mutations in the genesis of such a phenotype requires further investigation.

Angiogenesis is a complex process and its full understanding will require analysis at the level of morphology and gene expression. Here, we describe the poor prognosis associated with GMP, which is a highly characteristic lesion resulting from a gene expression profile that is as yet undefined. As antiangiogenic therapy is currently under intense investigation, it will be important to establish whether the presence of GMP alters the effectiveness of such therapies.
